# A Functional Polymorphism in B and T Lymphocyte Attenuator Is Associated with Susceptibility to Rheumatoid Arthritis

**DOI:** 10.1155/2011/305656

**Published:** 2011-02-22

**Authors:** Mie Oki, Norihiko Watanabe, Takayoshi Owada, Yoshihiro Oya, Kei Ikeda, Yasushi Saito, Ryutaro Matsumura, Yohei Seto, Itsuo Iwamoto, Hiroshi Nakajima

**Affiliations:** ^1^Department of Allergy and Clinical Immunology, Chiba University Hospital, Chuo-ku, Chiba 260-8670, Japan; ^2^Department of Rheumatology, Allergy, and Clinical Immunology, National Hospital Organization Chiba-East National Hospital, Chiba 260-8712, Japan; ^3^Research Center for Allergy and Clinical Immunology, Asahi General Hospital, Chiba 289-2511, Japan; ^4^Department of Molecular Genetics, Graduate School of Medicine, Chiba University, Chiba 260-8670, Japan

## Abstract

Inhibitory coreceptors are thought to play important roles in maintaining immunological homeostasis, and a defect in the negative signals from inhibitory coreceptors may lead to the development of autoimmune diseases. We have recently identified B and T lymphocyte attenuator (BTLA), a new inhibitory coreceptor expressed on immune cells, and we suggest that BTLA may be involved in the development of autoimmune diseases using BTLA-deficient mice. However, the role of BTLA in the pathogenesis of autoimmune diseases in humans remains unknown. We, therefore, examined the possible association between BTLA and rheumatoid arthritis (RA), systemic lupus erythematosus (SLE), and Sjögren's syndrome (SS) by conducting a case-control genetic association study. We found that 590C single-nucleotide polymorphism (SNP) of BTLA gene was significantly associated with susceptibility to RA, but not to SLE or SS. Furthermore, RA patients bearing this 590C SNP developed the disease significantly earlier than the patients without this allele. We also found that BTLA with 590C allele lacked the inhibitory activity on concanavalin A- and anti-CD3 Ab-induced IL-2 production in Jurkat T cells. These results suggest that BTLA is an RA-susceptibility gene and is involved in the protection from autoimmunity in humans.

## 1. Introduction

The immune system has developed multiple mechanisms to prevent deleterious activation of T cells. One such mechanism is the intricate balance between positive and negative costimulatory signals delivered to T cells. The B7-1 (CD80)/B7-2 (CD86)—CTLA-4 pathway is the best-characterized inhibitory pathway for T cell suppression and tolerance [1, 2]. Another inhibitory pathway involves programmed death-1 (PD-1) [3], which interacts with PD-L1 (also known as B7-H1) [4, 5] and PD-L2 (B7-DC) [6, 7]. Since these inhibitory coreceptors inhibit proliferation and cytokine production of T cells *in vitro* and *in vivo*, they are thought to play important roles in maintaining immunological homeostasis.

A defect in the negative signals from inhibitory coreceptors may reduce the threshold of autoreactive lymphocyte activation and, thus, may lead to the development of autoimmune diseases. This notion has been evidenced by the autoimmune phenotype or lymphocyte hyperreactivity in genetically manipulated mice that lack CTLA-4 and PD-1 [8–10]. In humans, CTLA-4 and PD-1 have been demonstrated to be involved in the regulation of autoimmune diseases by single nucleotide polymorphisms (SNPs) case-control association studies. It has been demonstrated that SNPs in PD-1 [11–13], but not in CTLA-4 [14], are associated with susceptibility to systemic lupus erythematosus (SLE) and rheumatoid arthritis (RA). On the other hand, SNPs in CTLA-4 are associated with disease risk in Grave's disease, autoimmune hypothyroidism, and type I diabetes [15].

We have identified B and T lymphocyte attenuator (BTLA) as an inhibitory coreceptor expressed on Th1 cells and B cells in mice [16]. Subsequently, analyses using anti-BTLA antibody have revealed that BTLA is expressed not only on CD4^+^ T cells and B cells but also on a wide range of hematopoietic cells including CD8^+^ T cells, NKT cells, NK cells, macrophages, and dendritic cells at various levels [17–20]. BTLA contains a single extracellular immunoglobulin (Ig) domain, a transmembrane region, and an intracellular region. There are two immunoreceptor tyrosine-based inhibitory motifs (ITIMs) in the intracellular region, and both of them are involved in the association with SHP-1 and SHP-2 [16, 21]. The ligand for BTLA is the TNF receptor family member herpesvirus entry mediator (HVEM) [22–24], which is broadly expressed on hematopoietic cells, including T cells, macrophages, and dendritic cells (DCs) [22]. Ligation of BTLA induces its tyrosine phosphorylation and SHP-1/SHP-2 association and then attenuates IL-2 production and proliferation of T cells [16, 21]. These findings suggest that BTLA functions as an inhibitory coreceptor through the interaction with HVEM and that HVEM-BTLA interaction may play a role in the prevention of autoimmune diseases.

We have shown that BTLA-deficient (BTLA^−/−^) mice exhibit increased specific antibody responses and enhanced sensitivity to experimental autoimmune encephalomyelitis [16]. In addition, we have recently shown that aged BTLA^−/−^ mice spontaneously develop autoantibodies and autoimmune hepatitis-like disease with lymphocytic infiltration in multiple organs [25]. Moreover, it has recently been demonstrated that SNP of BTLA is associated with RA susceptibility in the Taiwanese population [26]. However, the role of HVEM-BTLA pathway in the pathogenesis of autoimmune diseases in humans is still largely unknown.

We, therefore, attempted to identify SNPs in the human BTLA gene in the Japanese population and investigated its association with susceptibility to autoimmune diseases. We discovered two SNPs in the coding region of human BTLA gene in the Japanese population. We also found that 590C SNP was associated with susceptibility to RA but not to SLE or Sjögren's syndrome (SS). Furthermore, RA patients bearing this 590C SNP developed the disease significantly earlier than the patients without this allele. We also found that BTLA with 590C SNP lacked the inhibitory activity on Jurkat T cells. Our results suggest that dysfunction of BTLA is involved in the pathogenesis of RA.

## 2. Materials and Methods

### 2.1. Patients and Controls

A case-control genetic association study was conducted to examine the association between the BTLA gene and the development of RA, SLE, and SS. Patients with RA (81 patients, 80.2% women), SLE (64 patients, 95.3% women), and SS (60 patients, 95.0% women) were recruited into this study from Chiba University Hospital and Chiba-East National Hospital. All patients fulfilled the American College of Rheumatology revised criteria for RA [27] or SLE [28] and the Ministry of Health and Welfare criteria (1999 revision, Japan) for SS [29]. The ethics committee of the faculty of Chiba University approved this study. Control subjects were 71 unrelated healthy Japanese blood donors from the same geographic area (91.5% women).

### 2.2. Patient Profiles

The titers of rheumatoid factor (RF), matrix metalloproteinase-3 (MMP-3), C-reactive protein (CRP), erythrocyte sedimentation rate (ESR), antinucleotide antibody (ANA), immunoglobulin G (IgG), and complement 3 (C3) in sera of the patients were investigated at the first visit to the hospital. Age of disease onset was determined according to medical records.

### 2.3. Screening for Mutations in the Open Reading Frame of BTLA cDNA

Peripheral blood mononuclear cells (PBMCs) were isolated from 20 healthy Japanese donors by Ficoll-Paque (Amersham Biosciences, Uppsala, Sweden) density gradient centrifugation. Total RNA was purified from PBMCs using Isogen (Nippon gene, Toyama, Japan). First strand of cDNA was synthesized by a First-Strand Beads kit (Amersham Pharmacia biotech, Piscataway, NJ), and cDNA of BTLA was amplified using Pyrobest DNA polymerase (TaKaRa, Otsu, Japan). ExoSAP-IT-treated PCR products (USB, Cleveland, OH) were sequenced using a Big-Dye Terminators sequencing kit (Applied Biosystems, Foster City, CA) on an ABI PRISM 3100 Avant genetic analyzer (Applied Biosystems) according to the manufacturer's instruction. Derived sequences were compared with reported human BTLA cDNA sequences (DM004104 and NW_001838881.2) in the National Center for Biotechnology Information (NCBI) database.

### 2.4. SNP Genotyping

 PBMCs were treated with proteinase K and genomic DNA was purified by phenol-chloroform extraction. To amplify the BTLA gene encompassing the #590 or #800 SNP, PCR was performed using two sets of primers; exon4S 5′-TCCCTCCCCTTCCTTTTAGA-3′ and exon4AS 5′-AATAATGCCTGGCACATGGT-3′ were used for the amplification of exon 4 (for 590A/C), and exon5S 5′-TACCATGGCCGTAAGTGTCA-3′ and 3UT 5′-GAGCCCAGACAATGATGTCA-3′ were used for the amplification of exon 5 (for 800T/C). PCR products were then directly sequenced as described above. Heterozygous genotype was determined by identification of overlapping two short peaks of each nucleotide in the sequencing data.

### 2.5. Cell Culture and Infection of Jurkat T Cells

Jurkat T cells were maintained in RPMI 1640 medium supplemented with 10% fetal calf serum and antibiotics. Human BTLA gene with 590A or 590C was amplified from cDNA derived from a donor who carries the 590A or 590C using Pyrobest DNA polymerase with the following primers; 5′-GAAGATCTTTTTTCCATCACTGATATGTGC-3′ and 5′-CCGCTCGAGTCCCTGTTGGAGTCAGAAAC-3′. PCR products were isolated, digested with Bgl II and Xho I, and ligated into the multiple cloning site of retroviral expression vector Tb-lym-GFP RV [30], which contains an internal ribosome entry site (IRES) between the multiple cloning site and the green fluorescent protein (GFP) to generate 590A BTLA-IRES-GFP or 590C BTLA-IRES-GFP. Cloned vectors were transfected into amphotropic Phoenix packaging cells using FuGENE 6 transfection reagent (Roche, Indianapolis, IN). Forty-eight hours after transfection, viral supernatants were harvested and used for infection to Jurkat T cells in RetroNectin-coated plates (TaKaRa). Seven days after infection, Jurkat T cells expressing GFP were sorted by FACS Aria cell sorter (Becton-Dickinson, San Jose, CA). After two sequential rounds of cell sorting, over 95% of the cells were positive for GFP with similar levels of GFP intensity. Retrovirus-infected Jurkat T cells were stained with PE-conjugated antihuman BTLA mAb (MIH26) (BioLegend, San Diego, CA) for 60 min on ice. After washing, cells were analyzed on a FACSCalibur (Becton Dickinson) using CellQuest Pro software (Becton Dickinson).

### 2.6. IL-2 Production Assay

Jurkat T cells (5 × 10^5^ cells) that express 590C BTLA or 590A BTLA were stimulated with various concentrations of concanavalin A (ConA) (6.25–50 *μ*g/mL) in flat-bottomed plates that were coated with 2 × 10^5^ of HVEM-expressing CHO cells (a kind gift from Dr. K. M. Murphy, Washington University School of Medicine, St. Louis, MO) [23] to costimulate BTLA on Jurkat T cells. In other experiments, Jurkat T cells expressing 590C BTLA or 590A BTLA were stimulated with immobilized anti-CD3Ab (OKT3, 0.2 *μ*g/mL, BD PharMingen, San Diego, CA) and various concentrations of immobilized anti-BTLA Ab (MIH26, 0–20 *μ*g/mL, eBioscience, San Diego, CA) in the presence of anti-CD28 Ab (CD28.2, 1 *μ*g/mL, BD PharMingen). Twenty-four hours later, the culture supernatants were harvested and IL-2 concentration was measured by a human IL-2 ELISA kit (Biosource, Camarillo, CA) following manufacturer's instruction.

### 2.7. Statistical Analysis

The accord of SNP genotype distributions in each population with Hardy-Weinberg equilibrium was evaluated using chi-square goodness-of-fit test. The distributions of SNPs were compared between patients with RA, SLE, or SS and healthy controls by contingency table analysis and chi-square test. Relative risk was calculated as the ratio of incidence rates for 590A/A versus 590C SNP. Clinical features of RA patients were compared between the patients who carry 590A/A or 590C SNP using two-tail Mann-Whitney's *U* test. *P-*values less than  .05 were considered to be significant.

## 3. Results

### 3.1. Discovery of SNPs in the Human BTLA Gene

To identify SNPs in human BTLA gene, we isolated BTLA cDNA from peripheral blood mononuclear cells (PBMCs) of healthy Japanese donors (*n* = 20) and determined the sequences of the open reading frame of BTLA cDNA. By comparing these sequences with reported human BTLA cDNA sequences (DM004104 and NW_001838881.2) in NCBI database, we discovered two sequence variations (#590 and #800) in human BTLA gene in Japanese population (Table 1). They are localized in intracellular region of BTLA and cause amino acid replacement (Table 1). Variations at #590 (A to C) and #800 (T to C) were found in 15% and 40%, respectively, (Table 1). On the other hand, we could not find any insertion or deletion in the open reading frame of BTLA cDNA. Thus, we focused on #590 and #800 SNPs of BTLA gene to determine whether these SNPs are associated with susceptibility to autoimmune diseases.

### 3.2. Association of 590C SNP of BTLA Gene with Susceptibility to Rheumatoid Arthritis

To determine whether #590 and #800 SNPs of BTLA gene are involved in the susceptibility to autoimmune diseases, we first established a method that distinguishes homozygous and heterozygous genotypes of these SNPs. Genomic PCR was performed to amplify the region flanking each SNP and the nucleotide (A or C for #590 and T or C for #800) was then determined by sequencing the PCR products directly.

We then examined the frequencies of these SNPs in patients with RA (*n* = 81), SLE (*n* = 64), and SS (*n* = 60) as well as in healthy controls (*n* = 71). The frequencies of these SNPs of BTLA gene in patients with autoimmune diseases and in healthy subjects are summarized in Table 2. All groups are in Hardy-Weinberg equilibrium (data not shown), indicating that these genotyping data are reliable. Importantly, the frequency of individuals who carry 590 A/C or C/C genotype was significantly increased in RA patients but not in SLE patients and SS patients as compared with healthy controls (30.9% in RA, 14.1% in SLE, 15.0% in SS, and 14.1% in controls, Table 2). Statistical analysis revealed that the carriage of the 590A/C or C/C genotype was significantly increased in RA patients (*χ* ^2^ = 6.010, *P* = .014, relative risk = 2.19, 95% CI 1.13–4.24). We also found a significant association between 590C allele and RA susceptibility (*χ* ^2^ = 5.881, *P* = .015, relative risk = 2.28, 95% CI 1.14–4.56, Table 3). On the other hand, we could not detect any difference in the frequency of #800 SNP between RA patients and healthy controls (Tables 2 and 3). These results indicate that the carriage of 590C allele of BTLA gene is significantly associated with RA susceptibility and suggest that BTLA is involved in the pathogenesis of RA.

### 3.3. Correlation of 590C Allele of BTLA Gene with Early Onset of RA

**Figure 1:**
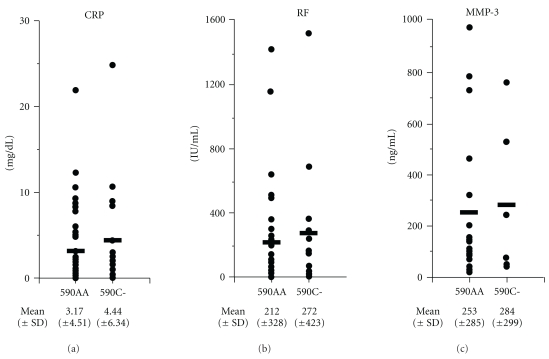
Disease profiles of rheumatoid arthritis patients with 590A/A and 590C SNPs of BTLA gene. Titers of C-reactive protein (CRP), rheumatoid factor (RF), and matrix metalloproteinase-3 (MMP-3) at the first visit to the hospital in RA patients with 590A/A and 590C (590A/C and C/C); SNPs of BTLA gene are shown.

We next compared disease profiles of RA patients who carry 590A/C or 590C/C genotype with those of RA patients who carry 590A/A genotype. RA patients with 590C allele developed the disease at significantly younger ages than the patients without this allele (590C-41.1 ± 17.1 years old versus 590A/A 49.9 ± 9.83 years old, *P* = .024), suggesting that 590C allele of BTLA gene accelerates the development of RA. We also examined the titers of C-reactive protein (CRP), rheumatoid factor (RF), and matrix metalloproteinase-3 (MMP-3) in sera of these RA patients at the first visit to the hospital (without medical treatment). However, the titers of these inflammatory parameters were not significantly different between RA patients with or without 590C allele (Figure 1).

### 3.4. BTLA with 590C Allele Loses the Inhibitory Function on ConA- or Anti-CD3-Induced IL-2 Production in Jurkat T Cells

**Figure 2:**
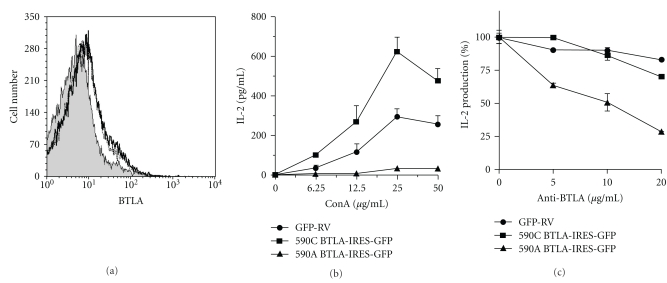
Loss of inhibitory function of 590C BTLA on ConA- and anti-CD3 Ab-induced IL-2 production in Jurkat T cells. (a) FACS analysis of BTLA expression in stable transfectants of Jurkat T cells with the retrovirus of 590A BTLA-IRES-GFP (solid line), 590C BTLA-IRES-GFP (dotted line), or GFP-RV (a negative control; shaded histogram). Both of transfectants express BTLA at similar levels. (b) ConA-induced IL-2 production from Jurkat T cells expressing 590A BTLA or 590C BTLA. Jurkat T cells expressing 590A BTLA or 590C BTLA (5 × 10^5^ cells) were cocultured with 2 × 10^5^ of HVEM-expressing CHO cells in the presence of various concentrations of concanavalin A (ConA). Twenty-four hours later, IL-2 levels in the supernatants were measured by ELISA. Representative data from three independent experiments are shown. (c) Anti-CD3 Ab-induced IL-2 production from Jurkat T cells expressing 590A BTLA or 590C BTLA. Jurkat T cells expressing 590A BTLA or 590C BTLA (5 × 10^5^ cells) were stimulated with immobilized anti-CD3 Ab (0.2 *μ*g/ml) and various concentrations of immobilized anti-BTLA Ab (MIH26) (0–20 *μ*g/ml) in the presence of anti-CD28 Ab (1 *μ*g/ml). Twenty-four hours later, IL-2 levels in the supernatants were determined by ELISA. Representative data of the percent IL-2 production from three independent experiments are shown.

To examine the basis of 590C SNP of BTLA gene for the susceptibility to RA, we compared the inhibitory activity of 590C BTLA with that of 590A BTLA *in vitro*. We employed Jurkat T cells to make stable transfectants because Jurkat T cells did not express any detectable BTLA mRNA even after activation (data not shown). Jurkat T cells were infected with retrovirus of 590C BTLA-IRES-GFP or 590A BTLA-IRES-GFP, and the infected cells were sorted with coexpressed GFP using FACS. As shown in Figure 2(a), the sorted Jurkat T cells expressed similar level of surface BTLA. These cells were then stimulated with various concentrations of concanavalin A (ConA) for 24 hours in the presence of HVEM-expressing CHO cells to costimulate BTLA simultaneously [23]. ConA induced IL-2 production from control virus (GFP-RV-) infected Jurkat T cells in a dose-dependent manner (Figure 2(b)). As expected, IL-2 production was strongly inhibited in cells expressing 590A BTLA (590A BTLA-IRES-GFP, Figure 2(b)). In contrast, in cells expressing 590C BTLA (590C BTLA-IRES-GFP), IL-2 production was rather enhanced as compared with control GFP-RV-infected cells (Figure 2(b)).

To further address the function of 590C BTLA, we stimulated Jurkat T cells expressing 590A BTLA or 590C BTLA with anti-CD3 Ab and various concentrations of immobilized anti-BTLA Ab. As expected, anti-BTLA Ab inhibited IL-2 production in cells expressing 590A BTLA in a dose-dependent manner (Figure 2(c)). In contrast, in cells expressing 590C BTLA or control GFP-RV-infected cells, anti-BTLA Ab did not significantly inhibit IL-2 production (Figure 2(c)). These results indicate that 590C BTLA lacks the inhibitory function on IL-2 production in Jurkat T cells and thus suggest that 590C SNP may be functionally associated with the susceptibility to RA.

## 4. Discussion

In this study, we show that BTLA is an RA-susceptibility gene and provide evidence that BTLA is involved in the protection from autoimmunity in humans. We found a significant association of 590C SNP of BTLA gene with susceptibility to RA, but not to SLE or SS by the case-control association study (Tables 2 and 3). We also found that the carriage of 590C allele of BTLA gene accelerated the development of RA. Moreover, we found that inhibitory activity of BTLA on ConA- and anti-CD3 Ab-induced IL-2 production in Jurkat T cells is lost by 590A to C conversion (Figures 2(b) and 2(c)). Taken together, these results suggest that 590C SNP is a polymorphism that lacks the inhibitory activity of BTLA and then increases the susceptibility to RA.

We show that a functional polymorphism at #590 on BTLA gene is associated with susceptibility to RA. Recently, associations between the polymorphisms of inhibitory coreceptors and autoimmune diseases have been reported. SNPs in CTLA-4 are associated with disease risk in Grave's disease, autoimmune hypothyroidism, and type I diabetes [15]. SNP in PD-1 is associated with susceptibility to SLE [11] and RA [12]. We show here that SNP in BTLA, the third member of inhibitory coreceptors, is associated with RA. Taken together, these findings suggest that dysfunction of inhibitory coreceptors is deeply involved in the pathogenesis of autoimmune diseases in humans.

We demonstrate that 590C BTLA lacks the inhibitory function on ConA- and anti-CD3 Ab-induced IL-2 production (Figures 2(b) and 2(c)). On the other hand, we found that there was no apparent difference in the surface levels of BTLA between transfectants of 590C BTLA and 590A BTLA (Figure 2(a)). Therefore, it is suggested that the alteration at #590 nucleotide with asparagine to threonine in the intracellular domain of BTLA may interfere with its signaling rather than its expression, presumably by downregulating the association of an undefined kinase that phosphorylates BTLA or SHP-1/SHP-2. This possibility needs to be tested by evaluating the ability of 590C BTLA protein to be phosphorylated or associated with SHP-1/SHP-2 in response to ligand-mediated activation.

We found that when Jurkat T cells expressing 590C BTLA were stimulated with ConA in the presence of HVEM-expressing CHO cells, these cells exhibited rather enhanced IL-2 production as compared with cells infected with control retrovirus (Figure 2(b)). In contrast, when Jurkat T cells expressing 590C BTLA were stimulated with immobilized anti-CD3 Ab in the presence of anti-BTLA Ab, these cells did not show enhanced IL-2 production (Figure 2(c)). In this regard, HVEM, a ligand for BTLA [23, 24], has been originally identified as a receptor for the TNF family members, LIGHT and LT*α* [31], and has been demonstrated to transmit a costimulatory signal in T cells through the interaction with LIGHT and LT*α* [32, 33]. Because it has been shown that HVEM and LIGHT are expressed on Jurkat T cells after TCR-mediated activation [34, 35] and because BTLA is suggested to form a ternary complex with HVEM and LIGHT and then may enhance LIGHT-HVEM signaling [24, 36], the expression of 590C BTLA not only lacks the function as an inhibitory coreceptor, but may enhance IL-2 production by this reverse costimulatory signaling through HVEM.

It is also possible that a change from asparagine to threonine in 590C BTLA may make BTLA a substrate of serine/threonine kinases and transduce stimulatory signals in association with HVEM, because sequence around threonine exhibits an XRXXT motif, which was reported to be phosphorylated by serine/threonine kinases such as Akt and CaMK II [37]. This possibility needs to be addressed by evaluating threonine phosphorylation of 590C BTLA in response to HVEM-mediated activation.

Many studies of multiple populations have supported a strong association between HLA class II and RA [38–41]. However, disease susceptibility regions for RA, other than HLA, are not yet completely understood. Genome-wide linkage study conducted by Cornélis et al. identified only one significant RA susceptibility locus which was located within chromosome 3q13 [42] and predicted that the candidate genes in this region might be CD80 and CD86. However, the association between RA and polymorphisms in CD80 and CD86 was not observed [43]. On the other hand, by taking a candidate gene approach, we could identify BTLA as a susceptibility gene for RA. Although the functional analyses of T cells expressing 590C BTLA or 590A BTLA in RA patients as well as in healthy controls are needed to reinforce our findings, we assume that a susceptibility gene for RA in chromosome 3q13 is BTLA [16].

Given that BTLA is associated with RA (Tables 2 and 3), we have explored in detail the arthritic changes in the extremities in aged BTLA^−/−^ mice. However, although BTLA^−/−^ mice spontaneously develop autoantibodies and autoimmune hepatitis-like disease with age [25], BTLA^−/−^ mice did not show any significant lesions resembling synovitis in RA patients (data not shown). The lack of RA-like phenotype in BTLA^−/−^ mice may be explained by the different expression pattern of BTLA in mice and humans in hematopoietic cell population [44, 45]. It is also possible that BTLA 590C may exhibit a costimulatory activity instead of a coinhibitory activity in some situations, and such activity may cause the difference in the arthritic changes in humans and mice.

We have shown that #590 SNP, but not #800 SNP, of BTLA gene is associated with RA susceptibility in the Japanese population (Tables 2 and 3). In contrast, Lin et al. have recently reported that SNP at #800 of BTLA gene is associated with RA susceptibility in the Taiwanese population [26]. They have reported that homozygous genotype (800 C/C or 800 T/T) of BTLA is the risk of RA comparing heterozygous genotype (800 T/C). The reason for this disparity is currently unknown, and further studies are required for addressing the disparity between these studies.

In conclusion, we have shown that the alteration of BTLA function by 590C SNP is involved in the pathogenesis of RA. Our data also give a new insight to the understanding of the pathogenesis of RA and may provide a clue to the application of BTLA for the treatment of RA.

## Figures and Tables

**Table 1:** Frequency of SNPs in BTLA gene in healthy Japanese donors.

Nucleotide no.	Exon	Original*	Mutant	Amino acid change	Frequency in Japanese
590	IV	A	C	Asn to Thr	15%
800	V	T	C	Leu to Pro	40%

SNPs in human BTLA gene were determined in healthy Japanese donors (*n* = 20) as described in Section 2.

*Nucleotide of human BTLA gene reported in NCBI database (DM004104 and NW_001838881.2).

**Table 2:** Association of 590A/C and 800T/C SNPs of human BTLA gene with susceptibility to autoimmune diseases.

	590A/A	590A/C	590C/C	*χ* ^2^	*P*	RR (95% CI)
Healthy subjects	61/71 (85.9%)	10/71 (14.1%)	0/71 (0.0%)			
RA	56/81 (69.1%)	24/81 (29.6%)	1/81 (1.3%)	6.010	.014	2.19 (1.13–4.24)
SLE	55/64 (85.9%)	9/64 (14.1%)	0/64 (0.0%)	0.000	.997	1.00 (0.43–2.30)
SS	51/60 (85.0%)	9/60 (15.0%)	0/60 (0.0%)	0.022	.882	1.07 (0.46–2.45)

	800T/T	800T/C	800C/C			

Healthy subjects	36/71 (50.7%)	29/71 (40.8%)	6/71 (8.5%)			
RA	45/87 (51.7%)	32/87 (36.8%)	10/87 (11.5%)	0.533	.766	0.98 (0.71–1.35)
SLE	35/56 (62.5%)	18/56 (32.1%)	3/56 (5.4%)	1.843	.398	0.76 (0.50–1.15)
SS	33/55 (60.0%)	16/55 (29.1%)	6/55 (10.9%)	1.885	.390	0.81 (0.54–1.21)

Genotyping of #590 and #800 SNPs of human BTLA gene was performed as described in Section 2.

RA: rheumatoid arthritis, SLE: systemic lupus erythematosus, SS: Sjögren's syndrome,

RR: relative risk, CI: confidence interval.

**Table 3:** Association of 590C allele of human BTLA gene with susceptibility to rheumatoid arthritis.

590	A Allele	C Allele	*χ* ^2^	*P*	RR (95% CI)
Healthy subjects	132	10			
RA	136	26	5.881	.015	2.28 (1.14–4.56)
SLE	119	9	0.000	.997	1.00 (0.42–2.38)
SS	111	9	0.020	.887	1.07 (0.45–2.53)

800	T Allele	C Allele	*χ* ^2^	*P*	RR (95%CI)

Healthy subjects	101	41			
RA	122	52	0.039	.844	1.04 (0.73–1.46)
SLE	88	24	1.822	.177	0.74 (0.48–1.15)
SS	82	28	0.364	.546	0.88 (0.58–1.33)

Same as the legend of Table 2.
